# *Nemopilema nomurai* jellyfish venom exerts an anti-metastatic effect by inhibiting Smad- and NF-κB-mediated epithelial–mesenchymal transition in HepG2 cells

**DOI:** 10.1038/s41598-018-20724-3

**Published:** 2018-02-12

**Authors:** Hyunkyoung Lee, Min Jung Pyo, Seong Kyeong Bae, Yunwi Heo, Indu Choudhary, Duhyeon Hwang, Hyeryeon Yang, Je-hein Kim, Jinho Chae, Chang Hoon Han, Changkeun Kang, Seungshic Yum, Euikyung Kim

**Affiliations:** 1 College of Veterinary Medicine, Gyeongsang National University, Jinju, 660-701 Republic of Korea; 2 Headquarters for Marine Environment, National Fisheries Research & Development Institute, Shiran-ri, Gijang-eup, Gijang-gun, Busan, 619-705 Republic of Korea; 3 Institute of Animal Medicine, Gyeongsang National University, Jinju, 660-701 Republic of Korea; 4 Gyeongnam Department of Environment & Toxicology, Korea Institute of Toxicology, Gyeongnam, 52834 Republic of Korea; 5 Marine Environmental Research and Information Laboratory, Gunpo, 15850 Korea; 6 South Sea Environment Research Center, Korea Institute of Ocean Science and Technology (KIOST), Geoje, Republic of Korea; 7 Faculty of Marine Environmental Science, University of Science and Technology (UST), Geoje, Republic of Korea

## Abstract

Epithelial–mesenchymal transition (EMT) is a key initial step in metastasis for malignant cancer cells to obtain invasive and motile properties. Inhibiting EMT has become a new strategy for cancer therapy. In our previous *in vivo* study, *Nemopilema nomurai* jellyfish venom (NnV) -treated HepG2 xenograft mice group showed that E-cadherin expression was strongly detected compared with non-treated groups. Therefore, this study aimed to determine whether NnV could inhibit the invasive and migratory abilities of HepG2 human hepatocellular carcinoma cells and to examine its effect on EMT. Our results revealed that transforming growth factor (TGF)-β1 induced cell morphological changes and downregulated E-cadherin and β-catenin expression, but upregulated N-cadherin and vimentin expression through the Smad and NF-κB pathways in HepG2 cells. Treatment of TGF-β1-stimulated HepG2 cells with NnV reversed the EMT-related marker expression, thereby inhibiting cell migration and invasion. NnV also significantly suppressed the activation of p-Smad3, Smad4, and p-NF-κB in a dose-dependent manner. These data indicated that NnV can significantly suppress cell migration and invasion by inhibiting EMT in HepG2 cells, and therefore might be a promising target for hepatocellular carcinoma therapeutics.

## Introduction

Recently, animal venoms have attracted the attention of researchers who are interested in identifying bioactive components and developing novel drug candidates because it has high sensitivity and specificity for target molecules^1^. Venom has long been used in traditional medicine, mainly in Asia and Africa^2–4^. For example, cobra venom has been used to treat joint pain, inflammation, and arthritis in Ayurveda, an Indian traditional medicine^5^. Bee venom has been used to treat chronic inflammation (rheumatoid arthritis), skin disease (acne and itch), and pain relief for thousands of years^6,7^. Various studies have also demonstrated that venoms of cnidarians (e.g., coral, hydra, jellyfish, and sea anemone) are abundant sources of enzymes, ion channel-regulatory peptides, and toxins with diverse actions^8–10^. Jellyfish venoms are considered as an interesting resource in the development of novel drugs for treating various diseases. *Aurelia aurita* venom has shown anticoagulant effect through strong fibrinogenolytic activity, by cleaving the Aα and Bβ chains of the fibrinogen molecule^11^. *Chiropsalmus quadrigatus* Haeckel venom has an active peptide with potential anti-angiotensin I-converting enzymatic activity^12^. *Nemopilema nomurai* is one of the largest jellyfish species and can grow up to a bell diameter of 2 m and weigh up to 200 kg. It is widely distributed in East Asian oceans near Korea, China, and Japan^13^. Several studies have reported that collagen extract from *N. nomurai* can stimulate the production of immunoglobulins and cytokines without any allergic complications, indicating that it has a regulatory effect on the immune system^14^. Qniumucin, a glycoprotein derived from *N. nomurai* jellyfish, has been found to have potential disease-modifying effects through the degeneration of articular cartilage in an *in vivo* osteoarthritis model^15^.

Hepatocellular carcinoma (HCC), one of the most common malignancies worldwide, causes cancer-related mortality^16^. Although diagnostic techniques and therapies for HCC are being continuously developed, mortality remains very high in patients with HCC owing to high recurrence and metastasis^17^. In general, metastasis involves multiple steps, including epithelial–mesenchymal transition (EMT), migration, matrix degradation, invasion into lympho-vascular tissue, extravasation, adhesion, and mesenchymal–epithelial transition (MET)^18^. To obtain invasive ability in early metastasis, EMT is an essential process that epithelial cells use to transform from an epithelial to a mesenchymal phenotype, with remarkable morphologic alterations. This is accompanied by decreased expression of epithelial markers (E-cadherin and β-catenin) and increased expression of mesenchymal markers and adhesion proteins (N-cadherin, vimentin, and fibronectin)^19^. Activation of EMT results in the loss of cell-cell adhesion of epithelial cancer cells. Actin cytoskeleton reorganization mediated by E-cadherin repression enables these cancer cells to migrate and invade into the bloodstream^18^. Therefore, EMT regulation plays an important role in the initiation and completion of metastasis.

Transforming growth factor (TGF)-β is one of the key mediators that initiates the EMT process^20^. TGF-β stimulates multiple pathways, including the classic Smad-dependent pathway and the alternative nuclear factor κB (NF-κB) pathway^20–23^. TGF-β activates the TGF-βI/II receptor, which phosphorylates Smad2 and Smad3, leading to the formation of a heteromeric Smad complex with cytosolic Smad4^24^. The Smad complex translocates to the nucleus where it regulates gene transcription by binding to Smad-binding elements in the promoters of target genes^25^. Recent studies have revealed that several transcription factors, including Snail, Slug, ZEB1, and SIP, are involved in EMT induction. When these transcription factors are overexpressed in cancer cells, they repress E-cadherin, leading to the induction and promotion of EMT^26^. In the alternative pathway, NF-κB might play a role in TGF-β-mediated EMT target gene induction^21,27^. TGF-β-activated kinase 1 (TAK1) can be activated by receptors for TGF-β, tumour necrosis factor α (TNF-α), and interleukin (IL)−1^27,28^. Activated TAK1 can phosphorylate the IκB kinase (IKK) complex (i.e., IKKα, IKKβ, and IKKγ/NEMO), which then phosphorylates IκBα. Degradation of IκBα results in the activation and translocation of p65 NF-κB^28^. Recent studies have demonstrated that EMT is associated with progression, poor prognosis, and chemo-resistance in HCC^29,30^. Accordingly, pharmacologic inhibition of EMT is considered an important treatment strategy to improve the prognosis for HCC patients.

Our previous study showed that *N. nomurai* venom (NnV) induces apoptotic cell death via dual inhibition of the phosphatidylinositol 3-kinase (PI3K)/Akt and mammalian target of rapamycin (mTOR) pathways in HepG2 human hepatocellular carcinoma cells and in a nude mouse model^31^. We also observed that tumour tissue in the NnV-treated group was more compact than that in the non-treated control group. Moreover, the NnV-treated group showed increased E-cadherin expression. Therefore, the objective of this study was to determine whether NnV can inhibit the invasion and migration abilities of HepG2 cells and to examine its effect on EMT.

## Results

### NnV blocks the migration and invasion of HepG2 cells

**Figure 1:**
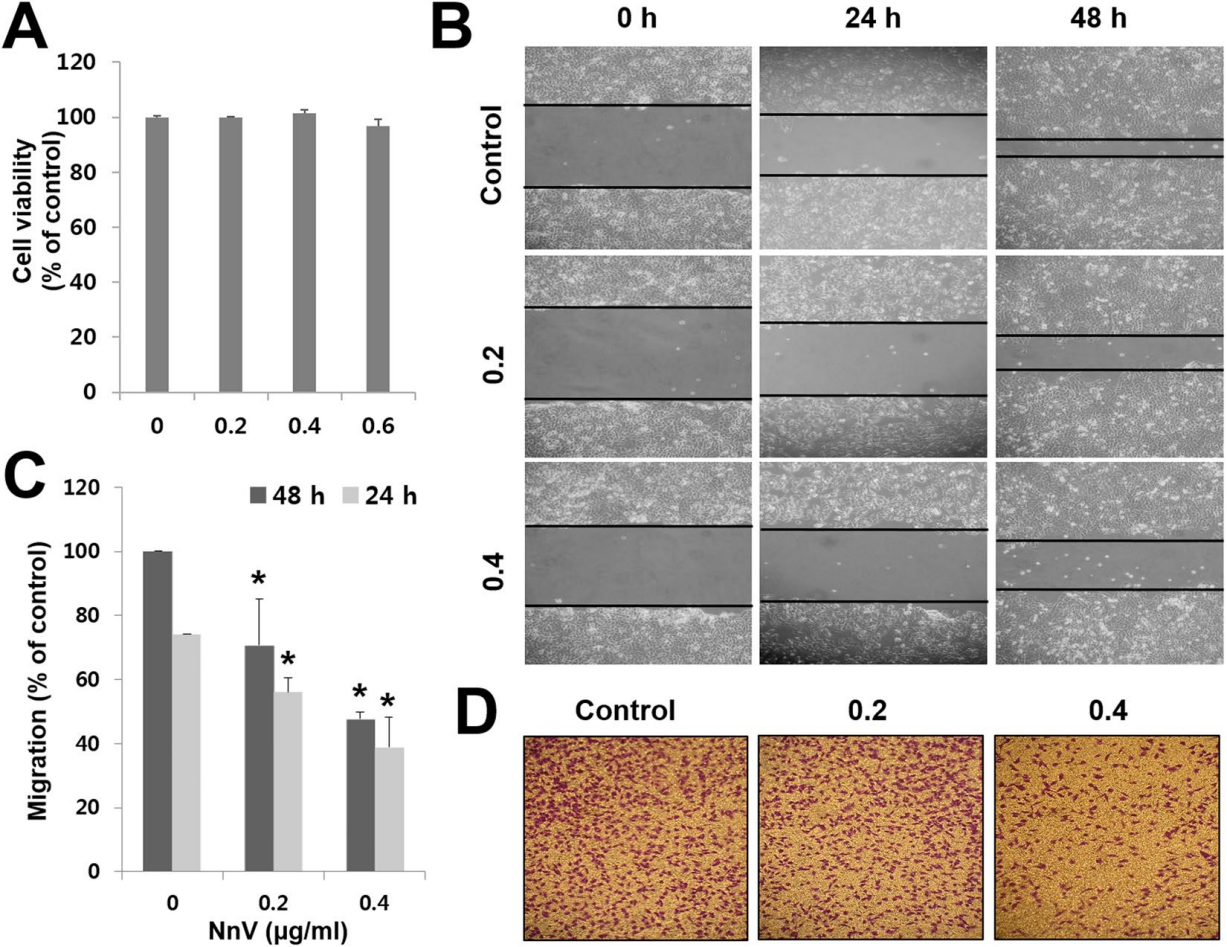
*Nemopilema nomurai* jellyfish venom (NnV) inhibits the migration and invasion of HepG2 cells. (**A**) Cells were plated in a 24-well cell culture plate and treated with various concentrations of NnV. Cell viability was measured by MTT assays. (**B**) Confluent monolayers of HepG2 cells were scratched with a pipette tip, treated with NnV, and photographed under a light microscope. (**C**) Migration activity was quantified with ImageJ software. (**D**) Invasion of HepG2 cells was measured using Boyden’s Transwell assays. Data are presented as the mean ± standard deviation (SD) from three observed fields. *p < 0.01 compared to the control.

To determine optimal concentrations of NnV without toxicity, cell viability was assessed by MTT assays. The results revealed that NnV at 0.6 µg/ml slightly inhibited the proliferation of HepG2 cells, whereas 0.2 and 0.4 µg/ml NnV had no significant effect (Fig. 1A). Therefore, the non-cytotoxic concentrations of NnV of 0.2 and 0.4 µg/ml were chosen for the following EMT experiments. As EMT is a key step in metastasis that initiates cancer cell migration, the migration potential of NnV-treated HepG2 cells was examined using wound-healing and transwell invasion assays. As shown in Fig. 1B and C, NnV significantly attenuated HepG2 cell migration in a concentration-dependent manner. The migratory ability of HepG2 cells treated with NnV at 0.2 and 0.4 µg/ml was reduced by 25% and 48%, respectively, at 24 h after treatment, and by 30% and 53%, respectively, at 48 h when compared with that of untreated control cells. Similarly, NnV inhibited HepG2 cell invasion in a concentration-dependent manner (Fig. 1D).

### NnV reverses EMT by downregulating Snail and Slug

**Figure 2:**
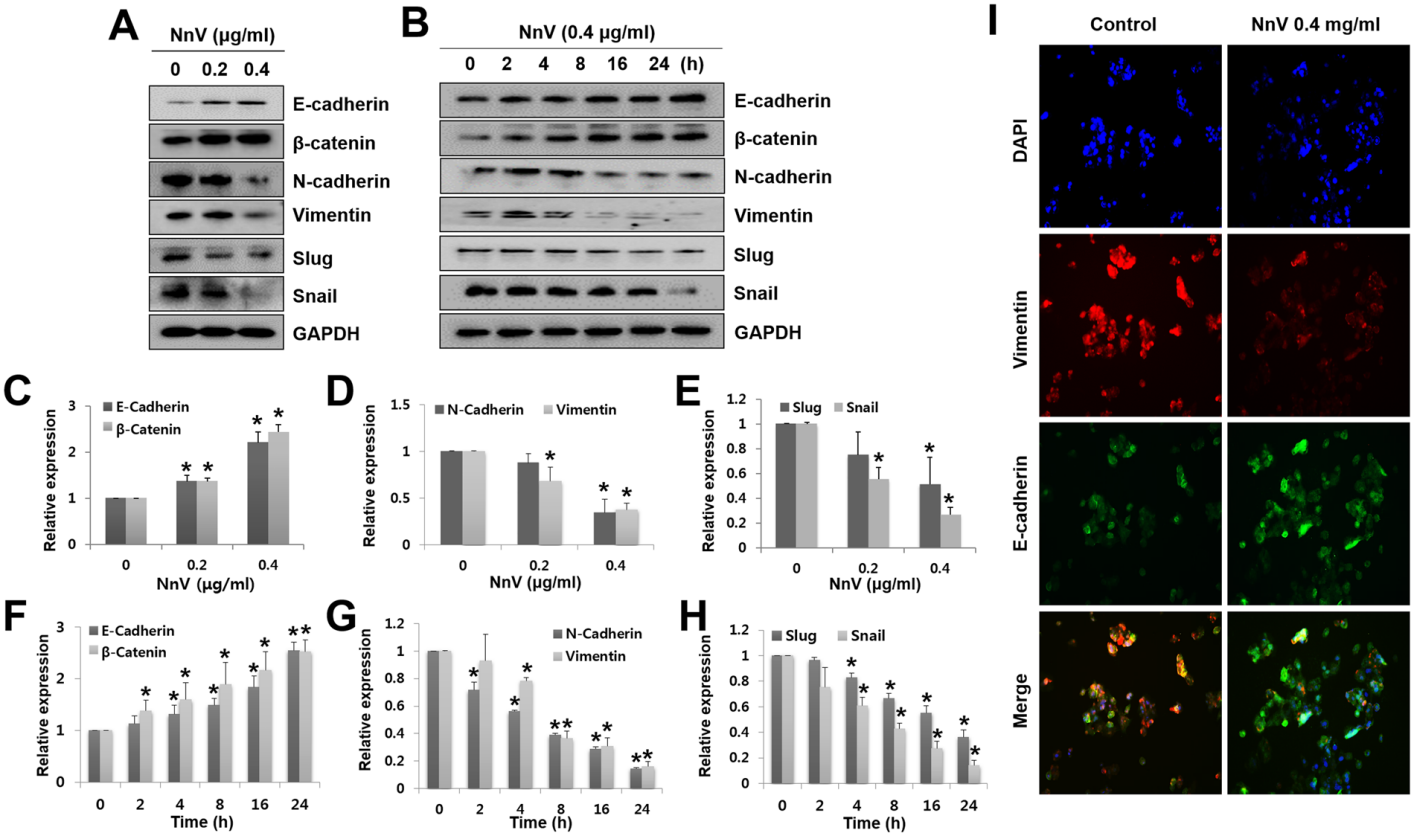
*Nemopilema nomurai* jellyfish venom (NnV) suppresses the expression of epithelial–mesenchymal transition (EMT)-related markers and transcription factors. Cells were treated with NnV at different doses (**A**) for different periods (**B**). (**C–H**) Expression levels of EMT markers and regulators were quantified using ImageJ. (**I**) Cells were treated with NnV, followed by immunostaining for E-cadherin (green) and vimentin (red), and counterstaining with DAPI (blue). Data are presented as the mean ± SD from three fields. *p < 0.01 compared to the control.

As EMT plays a critical role in accelerating migration and invasion, we next determined whether the inhibitory effects of NnV on migration and invasion are associated with EMT progression. Our previous study showed that NnV-treated tumours were less diffuse and had stronger E-cadherin expression than non-treated tumours. Similar to our *in vivo* data, NnV significantly increased the expression levels of the epithelial markers, E-cadherin and β-catenin, but extensively decreased those of the mesenchymal markers, N-cadherin and vimentin in both a concentration- and a time-dependent manner (Fig. 2A–H).

Snail and Slug are major transcriptional repressors of E-cadherin; therefore, we determined the expression levels of Snail and Slug in NnV-treated HepG2 cells. Interestingly, the expression levels of Snail and Slug were significantly attenuated in NnV-treated HepG2 cells in both concentration- and time-dependent manners. Furthermore, immunostaining analyses revealed that control cells more strongly expressed vimentin than E-cadherin, while NnV-treatment reversed the expression patterns of these markers (Fig. 2I). Based on these results, NnV inhibits the migration and invasion of HepG2 cells *via* suppressing EMT.

### NnV prevents TGF-β1-induced migration and invasion

**Figure 3:**
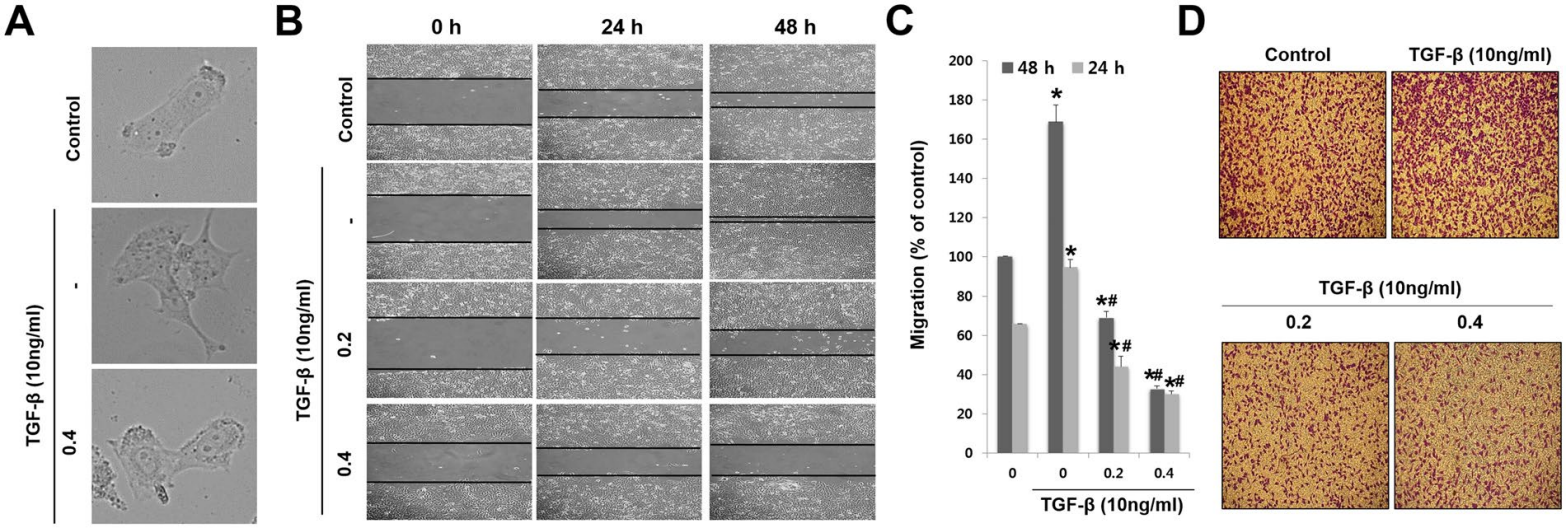
*Nemopilema nomurai* jellyfish venom (NnV) suppresses TGF-β-induced migration and invasion of HepG2 cells. (**A**) NnV reverses the morphological changes caused by TGF-β treatment. (**B**) Confluent monolayers of HepG2 cells were scratched with a pipette tip, treated with TGF-β and NnV, and photographed under a light microscope. (**C**) Migratory activity was quantified with ImageJ. (**D**) Invasion of HepG2 cells was assessed with Boyden’s Transwell assays. HepG2 cells were resuspended in serum-free Dulbecco’s modified Eagle’s Medium (DMEM; 5 × 10^4^ cells/200 µl) in the presence or absence of NnV (0, 0.2 and 0.4 µg/ml). TGF-β (10 ng/ml) was added to the lower chamber. Data are presented as the mean ± SD from three fields. *p < 0.01 compared to the control; #*p* < 0.01 compared to TGF-β group.

TGF-β1 can promote and maintain EMT during cancer progression^20^. Hence, we assessed whether NnV can abrogate TGF-β-induced cell migration and invasion of HepG2 cells. TGF-β1 treatment changed the morphology of HepG2 cells, showing elongated spindles and fibroblast-like nature (mesenchymal-type morphology). However, NnV-treated HepG2 cells showed less scattered and less elongated morphology (Fig. 3A). As shown in Fig. 3B and D the migration and invasion of HepG2 cells exposed to TGF-β1 were increased by 1.7-fold and 1.4-fold, respectively, as compared to those in control cells. However, NnV (0.2 and 0.4 µg/ml) treatment suppressed TGF-β1-induced cell migration and invasion by more than half when compared with TGF-β alone. These data demonstrate that NnV can attenuate the migration and invasion of HepG2 cells undergoing TGF-β1-induced EMT.

### NnV suppresses TGF-β1-induced EMT-related markers and transcriptional repressors

**Figure 4:**
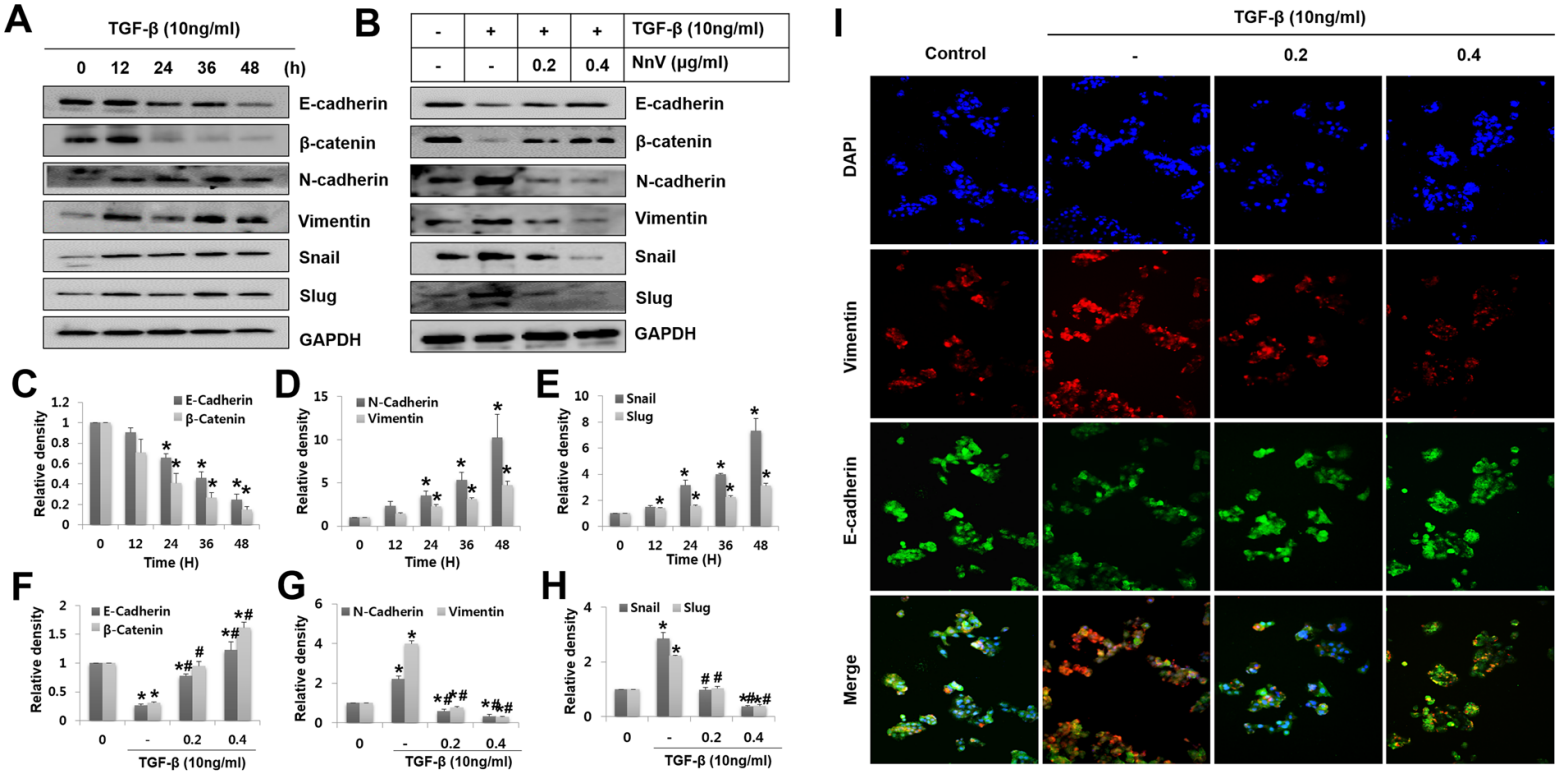
*Nemopilema nomurai* jellyfish venom (NnV) inhibits TGF-β-induced epithelial–mesenchymal transition (EMT) in HepG2 cells. HepG2 cells were treated with TGF-β or NnV, or both. (**A** and **B**) After 48 h of treatment, expression levels of EMT markers and regulators in cells were evaluated by western blotting. (**C–H**) The dose- and time-dependent expression levels of EMT markers and regulators were quantified in HepG2 cells treated with and without TGF-β or NnV. (**I**) The cells were immunostained for E-cadherin (green) and vimentin (red), and counterstained with DAPI (blue). Data are presented as the mean ± SD from five fields. *p < 0.01 compared to the control; #*p* < 0.01 compared to TGF-β group.

To further characterize the inhibitory effects of NnV on TGF-β1-induced migration and invasion in HepG2 cells, the cells were examined for the expression levels of EMT-related markers and repressors. To do this, HepG2 cells were pre-treated with 10 ng/ml of TGF-β for 24 h, then they were further incubated for additional 24 h in the absence or the presence of NnV at 0.2 and 0.4 µg/ml. TGF-β treatment alone significantly downregulated the epithelial markers, E-cadherin and β-catenin and significantly upregulated the mesenchymal markers, N-cadherin and vimentin. Concomitantly, it enhanced the expression of E-cadherin transcriptional repressors, Snail and Slug in a time-dependent manner (Fig. 4A). Whereas, NnV treatment considerably suppressed this switching of epithelial to mesenchymal markers (Fig. 4B), and completely inhibited TGF-β1-stimulated expressions of N-cadherin and vimentin as well as Snail and Slug. The immunostaining for E-cadherin and vimentin showed that TGF-β1-stimulated cells more strongly expressed vimentin than E-cadherin, whereas cotreatment with NnV and TGF-β1 reversed these expression of patterns (Fig. 4I).

### NnV decreases TGF-β1-induced canonical Smad pathway in HepG2 cells

**Figure 5:**
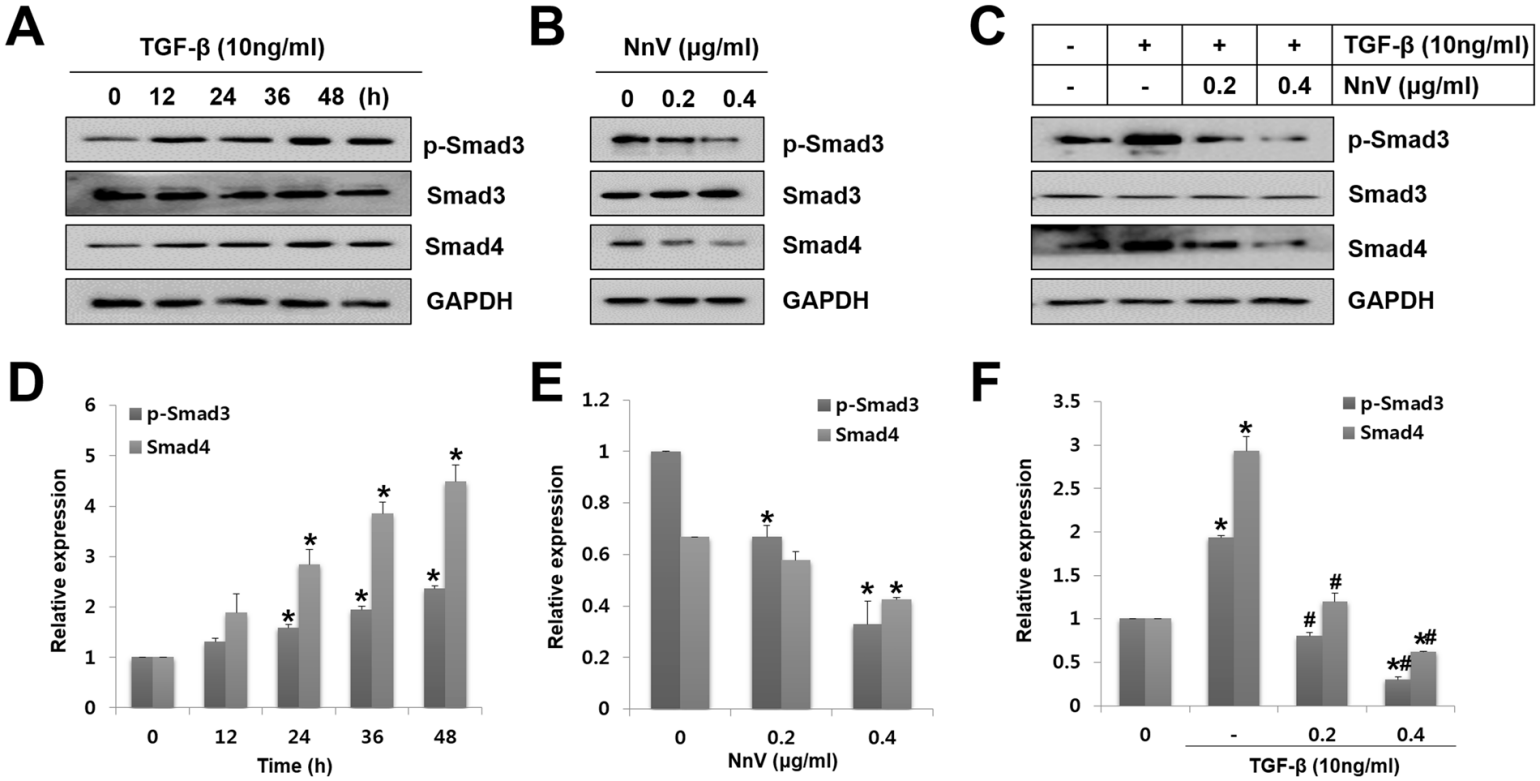
Role of *Nemopilema nomurai* jellyfish venom (NnV) in TGF-β-induced Smad pathway activation. (**A**) HepG2 cells were treated with TGF-β for various periods. The levels of p-Smad3, Smad3, and Smad4 were measured by western blotting. (**B**) HepG2 cells were treated with NnV at different concentrations. The levels of p-Smad3, Smad3, and Smad4 were determined by western blotting. (**C**) HepG2 cells were treated with NnV alone or pre-treated with TGF-β followed by NnV treatment. p-Smad3, Smad3, and Smad4 were assessed by western blotting. (**D–F**) p-Smad3, Smad3, and Smad4 expression was quantified using ImageJ. Data are presented as the mean ± SD from three fields. *p < 0.01 compared to the control; #*p* < 0.01 compared to TGF-β group.

The Smad pathway plays an important role in canonical TGF-β1 signalling. Phosphorylated Smad2 and Smad3 form a complex with Smad4 that translocates to the nucleus upon TGF-β1-dependent receptor is activated^25^. Hence, they were determined for the expression levels of p-Smad3, Smad3, and Smad4. From this, TGF-β1 promoted the phosphorylation of Smad3 from 12 h after treatment and the level of p-Smad3 reached a maximum at 48 h of treatment and it had also time-dependently provoked Smad4 expression, consistent with the result of p-Smad3 (Fig. 5A,D). Meanwhile, NnV treatment alone effectively intercepted the activation of Smad3 and Smad4 (Fig. 5B). NnV also significantly blocked the upregulation of p-Smad3 and Smad4 induced by TGF-β1 (Fig. 5C). Collectively, the data indicate that NnV blocks TGF-β1-induced EMT by inhibiting the complex formation of Smad3 and Smad4.

### NnV suppresses TGF-β1-induced NF-κB activation in HepG2 cells

**Figure 6:**
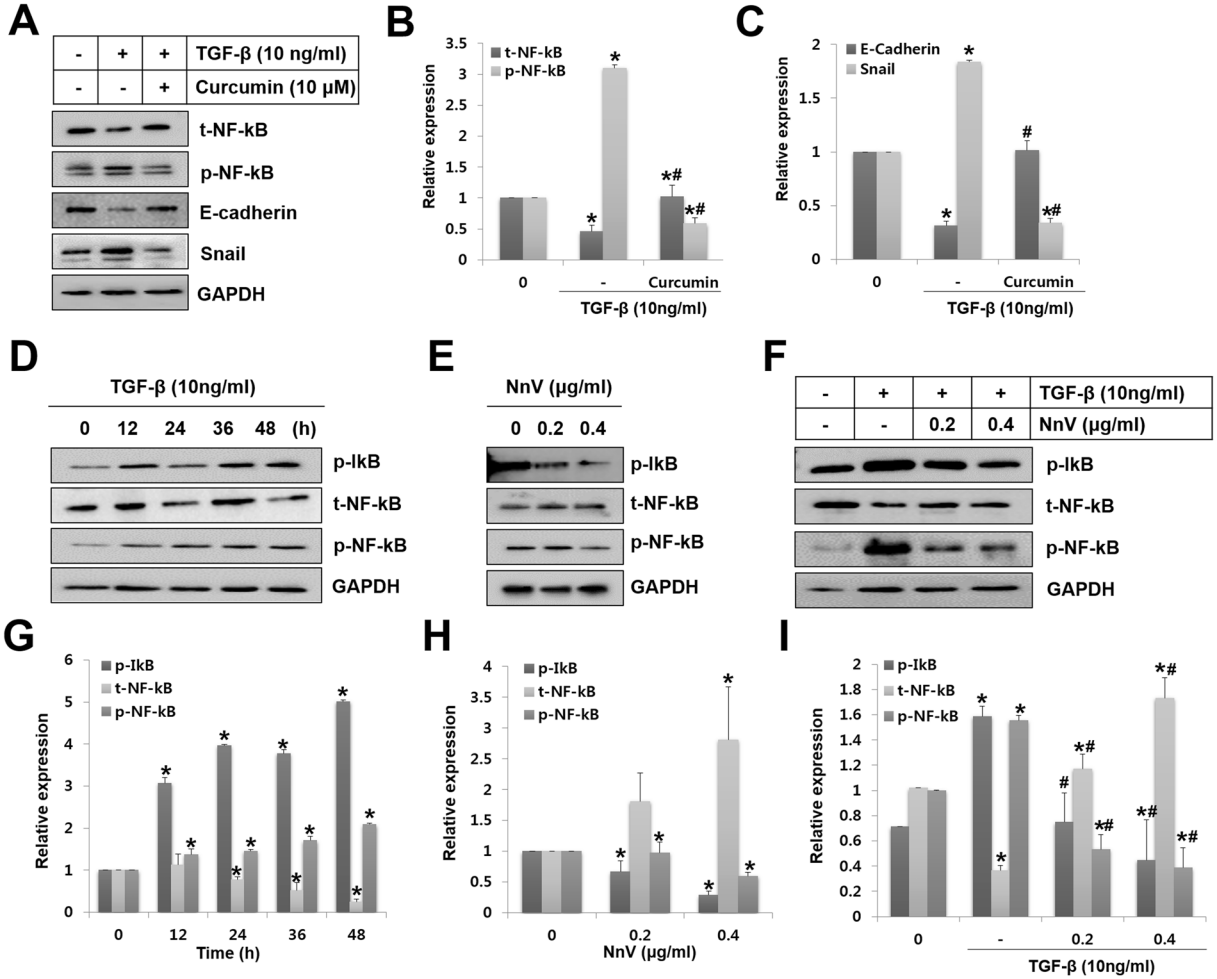
*Nemopilema nomurai* jellyfish venom (NnV) abrogates TGF-β-mediated E-cadherin downregulation through NF-κB inactivation. (**A**) HepG2 cells were pre-treated with curcumin (NF-κB inhibitor, 10 μM) followed by treatment with TGF-β for 48 h. Whole cell lysates were analysed for epithelial–mesenchymal transition (EMT) markers and NF- κB activation by western blotting. (**B** and **C**) t-NF-κB, p-NF-κB, E-cadherin, and Snail levels were quantified with ImageJ. (**D**) HepG2 cells were treated with TGF-β for various periods, and indicated proteins were detected by western blot analysis. (**E**) HepG2 cells were incubated with NnV for 48 h at different concentrations. The levels of p-IκB, NF-κB, and p-NF-κB were measured by western blotting. (**F**) HepG2 cells were treated with NnV alone or pre-treated with TGF-β followed by treatment with NnV. p-IκB, NF-κB, and p-NF-κB were assessed by western blotting. (**G**, **H**, and **I**) p-IκB, NF-κB, and p-NF-κB were quantified with ImageJ. Data are presented as the mean ± SD from three fields. *p < 0.01 compared to the control; #*p* < 0.01 compared to TGF-β group.

Numerous studies have reported that NF-κB pathway activation is associated with the response to TGF-β1^27,28^. To determine whether NF-κB is essential for TGF-β1-mediated EMT in HepG2 cells, curcumin was used to inhibit the TGF-β1-induced phosphorylation of NF-κB. Western blot analysis revealed that curcumin significantly inhibited NF-κB pathway induction by TGF-β1. It decreased the phosphorylation of NF-κB and Snail expression and increased the expression of total (t-)NF-κB and E-cadherin (Fig. 6A–C). These data suggest that the NF-κB pathway plays a role in the regulation of TGF-β1-mediated EMT in HepG2 cells.

Additionally, we examined the activation of NF-κB in HepG2 cells at various time points after TGF-β1 stimulation. The phosphorylation level of IκB was dramatically increased as of 12 h up to 48 h after induction with TGF-β1. In the cytosol, t-NF-κB decreased at 24 h after TGF-β1 stimulation. However, phosphorylation of NF-κB was increased from 12 h to 48 h (Fig. 6D,G). On the other hand, NnV treatment alone significantly suppressed the activation of NF-κB through reducing p-IκB and p-NF-κB, while it enhanced t-NF-κB (Fig. 6E,H). Furthermore, NnV significantly blocked TGF-β1-induced activation of NF-κB (Fig. 6F,I). These data suggest that inhibition of the NF-κB pathway plays an important role in TGF-β1-mediated EMT in HepG2 cells.

## Discussion

Our previous study for the first time showed that NnV suppresses hepatic tumour growth both *in vitr*o and *in viv*o^31^. Especially, tumour tissues in NnV-treated animals were less diffuse (more compact) and showed stronger E-cadherin expression than non-treated animals. As NnV can affect cell-cell density and cell-cell matrix interactions, we examined its effects on migration and invasion in HepG2 cells for the current study. Interestingly, NnV blocked migratory and invasive behaviours of hepatocarcinoma HepG2 cells wich is not associated with its cytotoxic effect. We found that the inhibitory effects of NnV on migration and invasion were related with EMT regulation. E-cadherin, a transmembrane adhesion receptor, is known as a main marker of EMT. It attaches to α-, β-, and p120-catenins to interact with the intracellular actin filament network. Dissociation of these complexes contributes to the strength and integrity of cell-cell adhesions and the reorganization of the actin cytoskeleton, leading to migration and local invasion into the surrounding tissues^32^. During EMT, an E-cadherin-to-N-cadherin switch occurs (mesenchymal phenotype), thus facilitating migration and invasion^20^. Our data showed that NnV significantly increased the expression levels of E-cadherin and β-catenin in HepG2 cells. On the other hand, N-cadherin and vimentin expression levels in HepG2 cells were decreased by NnV in both concentration- and time-dependent manner. E-cadherin also inhibits the spreading of HCC cells into surrounding tissues and resistance to apoptosis. The increased E-cadherin and β-catenin expressions induced by NnV blocked the acquisition of invasive and migratory phenotypes by HepG2 cells, suggesting that NnV might be useful to enhance the efficiency of chemotherapeutics and prevent HCC occurrence at an early stage.

To better understand the mechanism underlying EMT inhibition by NnV, we have focused on the TGF-β signaling pathway, which is well known to plays a vital role in promoting EMT and tumour growth. TGF-β1 is primarily found in a latent form in the tissue microenvironment and is activated after proteolytic tissue remodeling^20^. Activated TGF-β induces a favourable microenvironment for tumour cell growth, and is the switch that moves cells toward malignant progression via EMT progression^33^. Recent clinical observations have shown that serum and urine TGF-β levels are higher in patients with HCC than in healthy controls and worsen prognosis. Hence, high TGF-β is regarded as a hallmark of HCC^34,35^ and one of the targets for developing therapeutic interventions for metastatic cancer. Our results showed that TGF-β1-mediated cell morphological changes were reversed from spindle shapes with created fiber (mesenchymal type) to clustered and polarized shapes (epithelial type) by NnV treatment in HepG2 cells. NnV also blocked the migratory and invasive behaviours of TGF-β1-stimulated HepG2 cells, and suppressed TGF-β1-mediated N-cadherin and vimentin expression as well as enhanced E-cadherin and β-catenin expression. These data support the NnV indeed targets EMT process in HepG2 cells which are treated with TGF-β.

One of the possible mechanisms for the inhibitory effect of NnV on EMT is that NnV suppresses the canonical TGF-β signaling pathway through Smad3 and Smad4. Smad3 and Smad4 play pivotal roles in TGF-β-mediated EMT, tumour progression, and metastasis in several cell types^36^. Smad4 knockdown potently inhibits TGF-β-induced EMT in NMuMG cells based on the morphologic transformation from epithelial- to fibroblast-like cells, formation of stress fibres, inhibition of E-cadherin expression, and enhanced expression of various mesenchymal markers^37^. In the present study, NnV inhibits Snail and Slug expressions by intercepting TGF-β1-induced p-Smad3 and Smad4, consequently recovering of epithelial marker expression (E-cadherin and β-catenin). TGF-β1 signalling is not limited to the Smad pathway. It can also be mediated by non-Smad pathways with impact on EMT progression. Recently, it was revealed that NF-κB activation study showed that TGF-β1 increased the levels of p-IκB and p-NF-κB. Interestingly, the present work showed that TGF-β1 increased the levels of p-IκB and p-NF-κB. However, the specific NF-κB inhibitor curcumin suppressed the NF-κB pathway and Snail expression, but increased E-cadherin expression. Taken together, NF-κB signalling seems to be an essential therapeutic target for hepatic tumours. NnV significantly suppressed TGF-β-mediated phosphorylation of IκB, which resulted in stabilization of the NF-κB-IκB interaction, thus preventing translocation of NF-κB into the nucleus and the induction of Snail, corroborating its therapeutic potential.

The present study for the first time demonstrated that NnV exhibits anti-metastatic activity in HepG2 cells through reversing EMT *via* the Smad and NF-κB pathways. Jellyfish venom is composed of various molecules including peptides, enzymes, neurotoxin, cytolysin, haemolysin with biological effects^38–46^. According to our previous study on the anti-cancer effect of NnV, metalloprotease component in NnV might be the main component contributing to apoptotic HepG2 cell death^31^. In support of this notion, other venoms (of snakes and spiders) also have metalloprotease activities that inhibit cell adhesion, proliferation, migration, invasion, and angiogenesis^47–50^. Jararhagin, a purified snake venom metalloprotease from *Bothrops jararaca*, inhibits cell adhesion and has cytotoxic effects on melanoma cells^47,49^. TSV-DM, a metalloprotease from *Trimeresurus stejnegeri*, inhibits cell proliferation and induces apoptosis in ECV304 cells^48^. Therefore, further studies on the identification and characterization of anti-metastatic components of NnV are needed. By any measure, this finding can be helpful for researchers in the discovery and development of new drug candidates derived from jellyfish venom.

## Materials and Methods

### Chemicals and reagents

DMEM, foetal bovine serum (FBS), bovine serum albumin (BSA), penicillin, streptomycin, and trypsin were purchased from Gibco-BRL (Grand Island, NY, USA). Dimethyl sulfoxide (DMSO), 3-(4,5-dimethylthiazol-2-yl)−2,5-diphenyltetrazolium bromide (MTT), and TGF-β were from Sigma-Aldrich (St. Louis, MO, USA). Antibodies for E-cadherin, β-catenin, N-cadherin, vimentin, Slug, Snail, p-Smad3, p-Smad3, Smad4, p-IκB, NF-κB, p-NF-κB, and GAPDH were obtained from Cell Signaling Technology (Beverly, MA, USA). All other reagents used were of the purest grade available.

### Jellyfish collection and preparation

Specimens of *N. nomurai* jellyfish were captured from the Korea Strait along the coast of Geoje in September 2012. Only tentacles were collected on ice and transferred immediately to the laboratory for further preparation. Nematocysts were isolated from the dissected tentacles as described by Bloom *et al*.^51^, with slight modification. In brief, dissected tentacles were rinsed with cold seawater to remove debris. They were then placed in three volumes of cold seawater for 24 h with gentle swirling for 1 h once daily at 4 °C. After autolysis at 4 °C for 24 h, the supernatant was collected and centrifuged at 4,000 × *g* for 10 min, and the settled was resuspended in fresh seawater and set for autolysis for 24 h. This process was repeated for 3 days. The sediments were collected and centrifuged at 4,000 × *g* for 10 min and washed several times with fresh distilled water by centrifugation 100 × *g* at 4 °C for 5 min, until the debris surrounding the nematocysts was mostly removed. Finally, the undischarged nematocysts were collected, lyophilized, and stored at −70 °C until use.

### Venom extraction and preparation

Venom was extracted from freeze-dried nematocysts using the technique described by Carrette and Seymour^52^, with minor modifications. In brief, venom was extracted from 50 mg of lyophilized nematocyst powder in 1 ml of cold phosphate-buffered saline (PBS, pH 7.4, 4 °C) using glass beads (0.5 mm in diameter) by ten cycles of shaking at 3000 rpm for 30 s with intermittent cooling on ice. The venom extract was transferred to an Eppendorf tube and centrifuged (15,000 × *g*) at 4 °C for 30 min. The supernatant was used as the NnV. The protein concentration of the NnV was determined using the Bradford method (Bio-Rad, CA, USA), and NnV dose was determined based on this protein concentration^53^.

### Cell culture

HepG2 cells were purchased from the American Type Culture Collection (ATCC). The cells were cultured in DMEM supplemented with 10% heat-inactivated FBS and 100 μg/ml penicillin-streptomycin-amphotericin B solution at 37 °C in a humidified atmosphere with 5% CO_2_.

### Cell viability

To determine potential cytotoxic effects of NnV on HepG2 cells, the cell viability was measured by MTT reduction assays in the absence or presence of NnV as previously described^45^. HepG2 cells were seeded in 24-well plates at 4 × 10^4^ cells/well and cultured for 24 h. Cells were treated with the indicated concentrations (0–0.6 μg/ml) of NnV for another 48 h. After incubation, MTT solution (5 mg/ml) was added to each well and incubated at 37 °C for 3 h. After the supernatant was removed, DMSO was added to each well to dissolve the formazan crystals generated. Cell viability was determined by measuring the optical density at 540 nm using an ELISA multiwell plate reader (PowerWaveXS; BioTek Instruments, Winooski, VT, USA).

### Wound healing assay

Cell migration assays were performed using 12-well plates, as previously described^54^. HepG2 cells were seeded in 12-well plates (2 × 10^5^ cells/well) and grown to 80–90% confluence. After aspiration of the medium, cells were scraped with a 200 µl sterile pipette tip to create a straight scratch. The cells were washed twice with PBS to remove cellular debris and then, serum-free DMEM was added. HepG2 cells were treated with TGF-β (10 ng/ml) and/or NnV (0, 0.2, and 0.4 µg/ml). Cell migration into the wound area was photographed at 0, 24, and 48 h. The level of cell migration was determined using a Hewlett-Packard scanner and ImageJ software (NIH, Bethesda, MD, USA), and was expressed as a percentage of the initial wound area. Duplicate wells for each condition were examined for each experiment, and each experiment was repeated three times.

### Invasion assay

The invasive behaviour of HepG2 cells was tested using a cell invasion chamber kit (BD Biosciences, San Jose, CA, USA). HepG2 cells were suspended in serum-free DMEM (5 × 10^4^ cells/200 µl) in the absence or the presence of TGF-β (10 ng/ml) and/or NnV (0, 0.2, and 0.4 µg/ml). The cells were seeded into the upper chamber on a Matrigel-coated filter, and 500 µl of DMEM containing 10% FBS was added to the lower chamber. After a 48 h incubation, the non-invaded cells were removed from the upper surface of the filter.The invading cells on the lower surface of the filter were stained with crystal violet for 15 min, rinsed with water, dried, and quantified under a light microscope.

### Western blot analysis

Cells plated on microplate dishes were incubated with various concentrations of NnV for the indicated periods and then washed with cold PBS. The treated cells were collected by scraping in RIPA buffer containing a protease inhibitor cocktail. Lysates were separated on a 12% SDS-polyacrylamide gel, transferred to PVDF membranes (Bio-Rad, Hercules, CA, USA), and immunoblotted with specific primary antibodies overnight at 4 °C. After washing, the membranes were incubated with a horseradish peroxidase-conjugated secondary antibody (Cell Signaling Technology, Beverly, MA, USA) for 1 h at room temperature. The blots were visualized with enhanced chemiluminescence (Amersham Biosciences, Buckinghamshire, UK) and analysed using a ChemiDoc XRS (Bio-Rad). Densitometry measurements were conducted with a Hewlett-Packard scanner and ImageJ.

### Immunofluorescence analysis

HepG2 cells were grown on cell culture slides (SPL Life Science, Pocheon, Korea) to 40–50% confluence (24 h). Then, the cells were treated with TGF-β (10 ng/ml) and/or NnV and washed with PBS for 5 min thrice. The cells were fixed with 4% paraformaldehyde for 10 min and blocked with 3% FBS for 1 h at room temperature. The cells were incubated with primary antibodies (anti-E-cadherin and anti-vimentin) overnight at 4 °C. Then, the slides were then washed and incubated with secondary FITC-conjugated antibody for 1 h at room temperature. The cells were washed and mounted on coverslips with mounting medium containing DAPI (Vector Laboratories, Burlingame, CA, USA).

### Statistical Analysis

The results are expressed as the mean ±SD. One-way analysis of variance (ANOVA) was used to compare means. *P* < 0.01 was considered significant.

## Electronic supplementary material

Supplementary information
