# Supplementary material for: Nemopilema nomurai jellyfish venom exerts an anti-metastatic effect by inhibiting Smad- and NF-κB-mediated epithelial–mesenchymal transition in HepG2 cells
Source: Sci Rep. 2018 Feb 12;8:2808. doi: 10.1038/s41598-018-20724-3 (PMC5809415; doi:10.1038/s41598-018-20724-3)
Supplement: Supplementary file 1 — Supplementary information [file 41598_2018_20724_MOESM1_ESM.pdf]

Nemopilema nomurai jellyfish venom exerts an anti-metastatic effect by inhibiting Smad- and NF-κB-mediated epithelial–mesenchymal transition in HepG2 cells

Hyunkyoung Lee[1,3], Min Jung Pyo[1], Seong Kyeong Bae[1], Yunwi Heo[4], Indu Choudhary[1], Duhyeon Hwang[3], Hyeryeon Yang[1], Je-hein Kim[4], Jinho Chae[5], Chang Hoon Han[2], Changkeun Kang[1], Seungshic Yum[6,7*], Euikyung Kim[1,3*]

Fig.2A

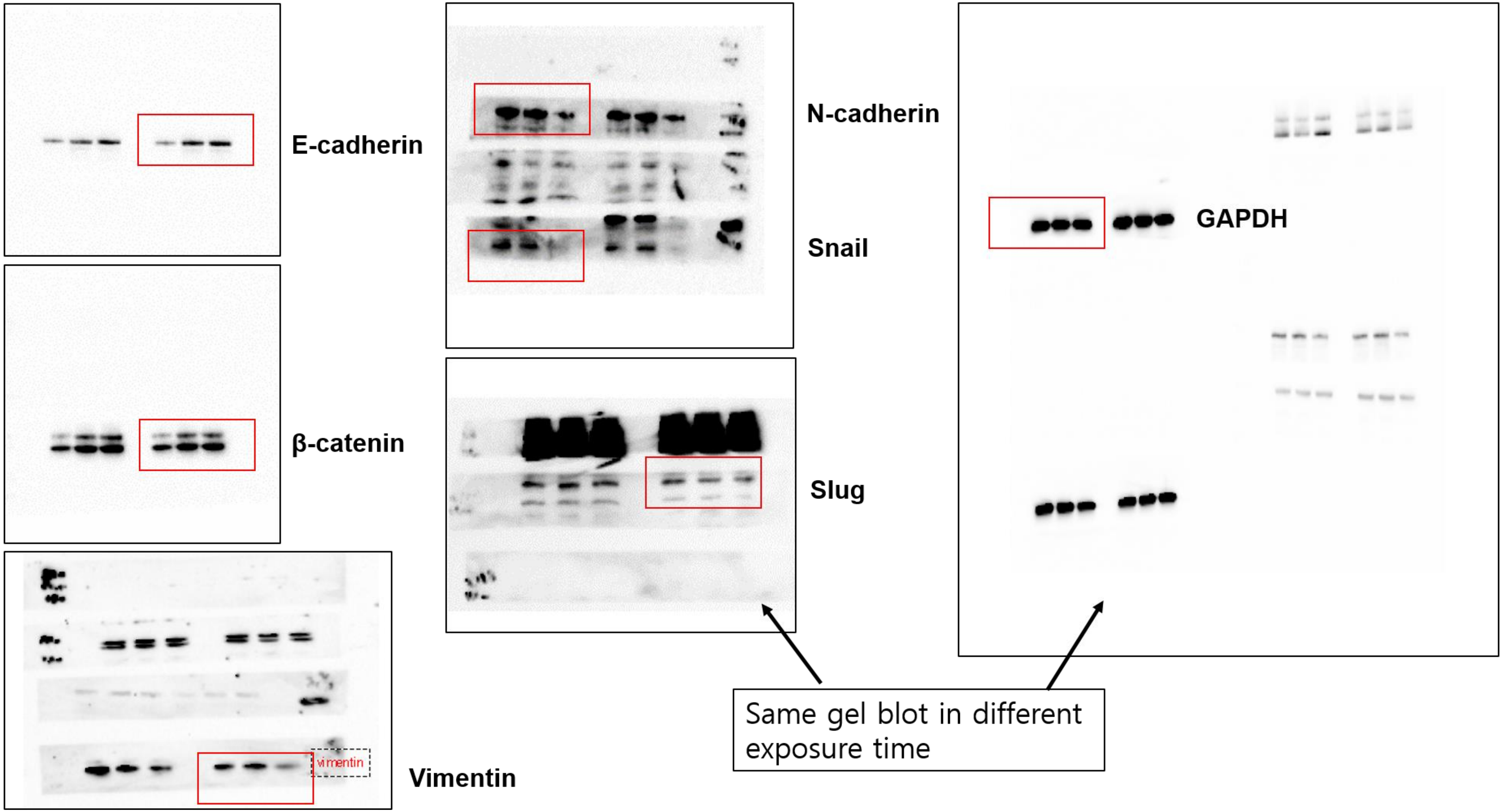

E-cadherin
β-catenin
Vimentin
vimentin
N-cadherin
Snail
Slug
GAPDH
Same gel blot in different exposure time

Fig.2B

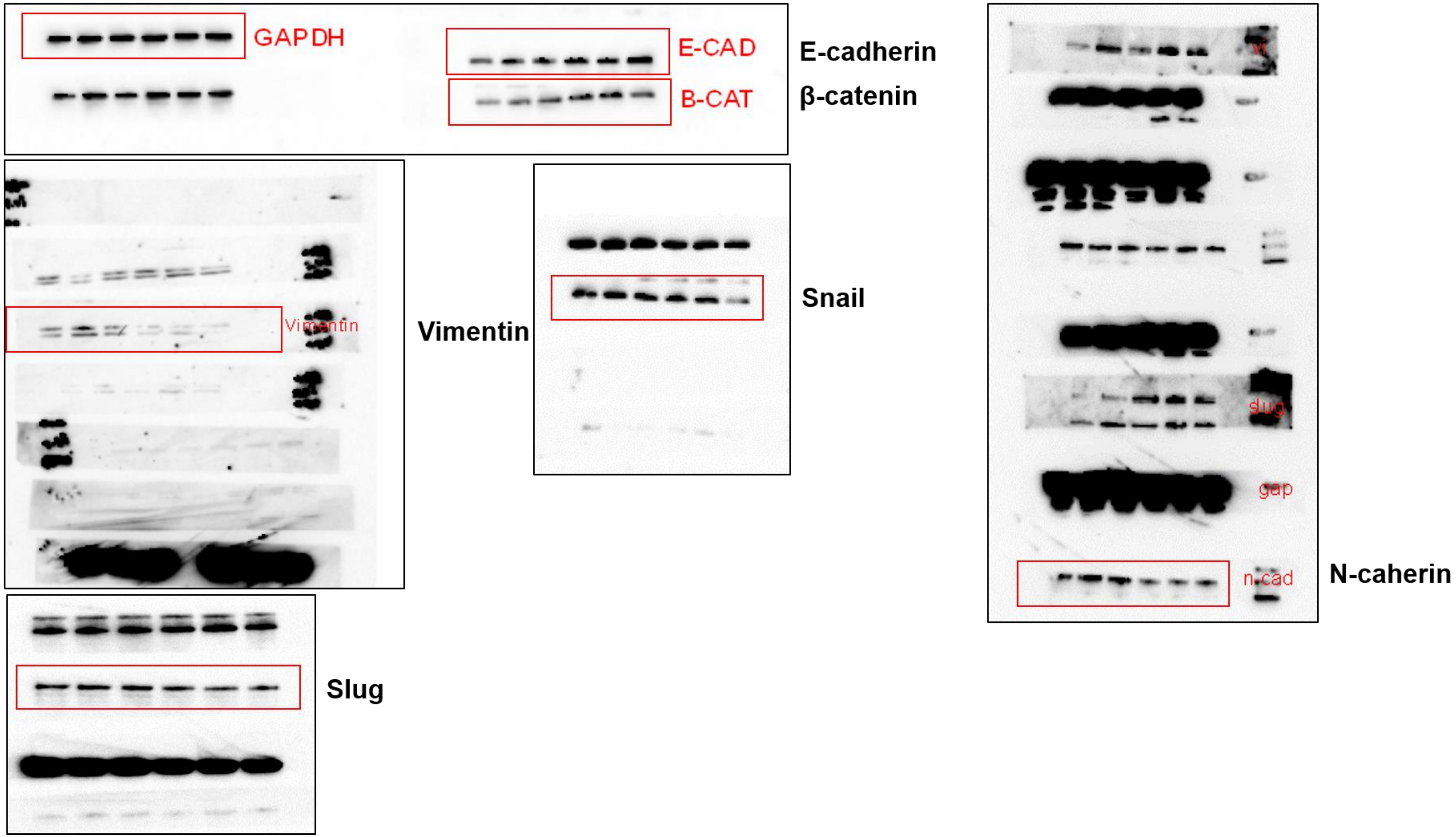
GAPDH
E-CAD
B-CAT
E-cadherin
β-catenin
Vimentin
Vimentin
Snail
Slug
slug
gap
n-cad
N-caherin

Fig.4A

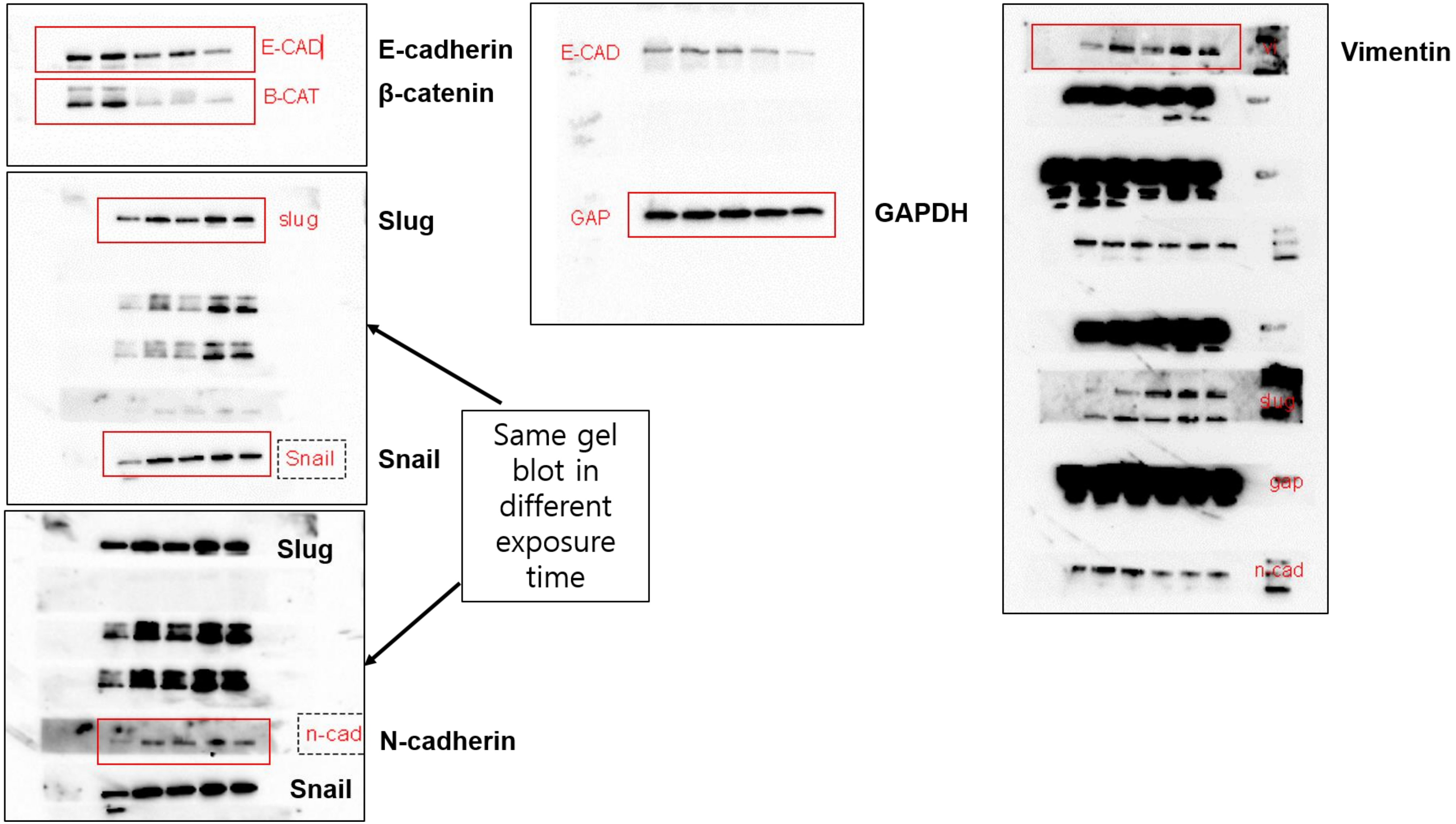
E-CAD
B-CAT
E-cadherin
β-catenin
E-CAD
GAP
GAPDH
Vimentin
slug
Slug
Snail
Snail
Same gel blot in different exposure time
Slug
n-cad
N-cadherin
Snail
slug
gap
n.cad

Fig.4B

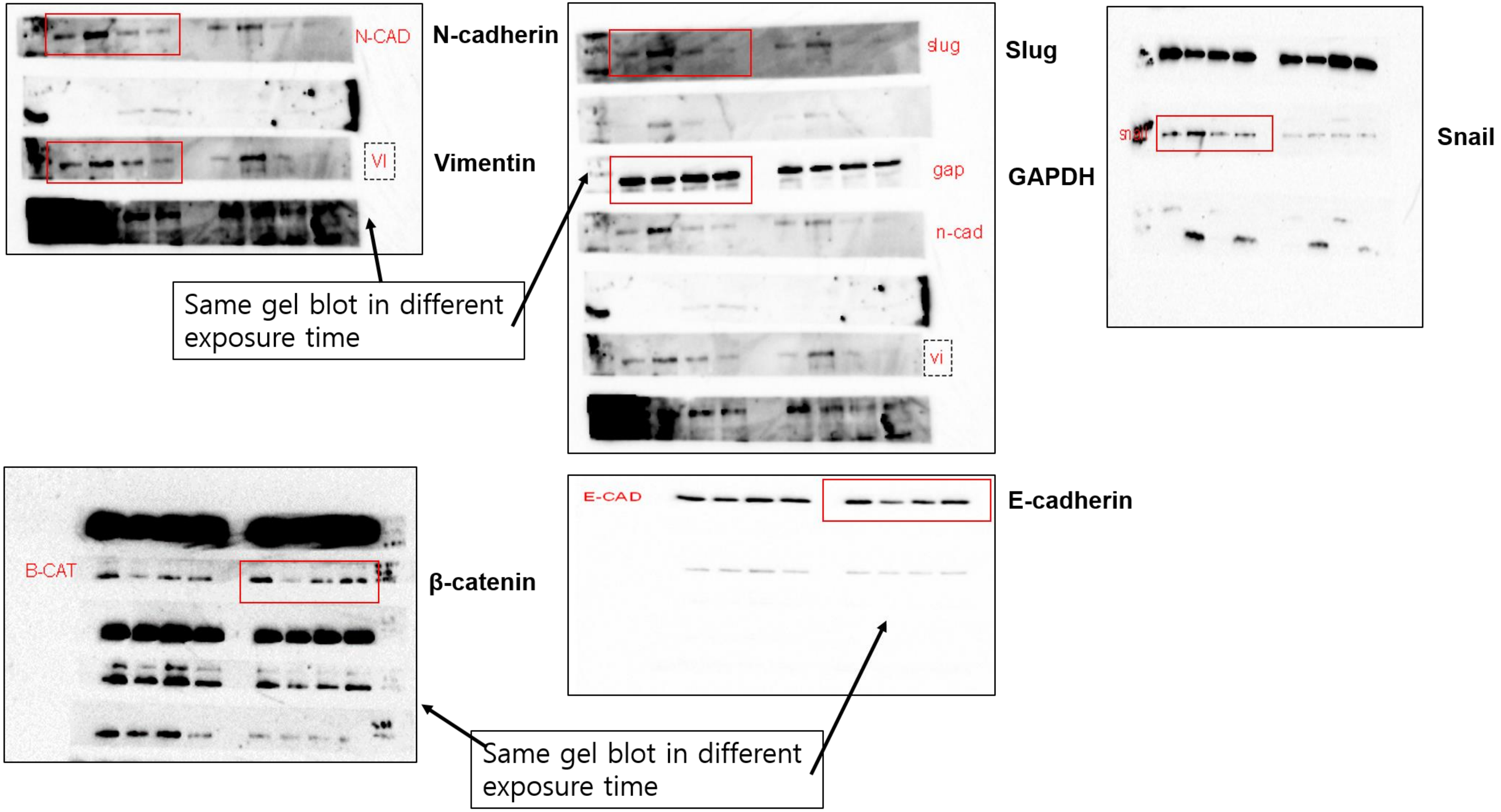
N-CAD
N-cadherin
VI
Vimentin
Same gel blot in different exposure time
slug
Slug
gap
GAPDH
n-cad
vi
snail
Snail
B-CAT
β-catenin
E-CAD
E-cadherin
Same gel blot in different exposure time

Fig.5A

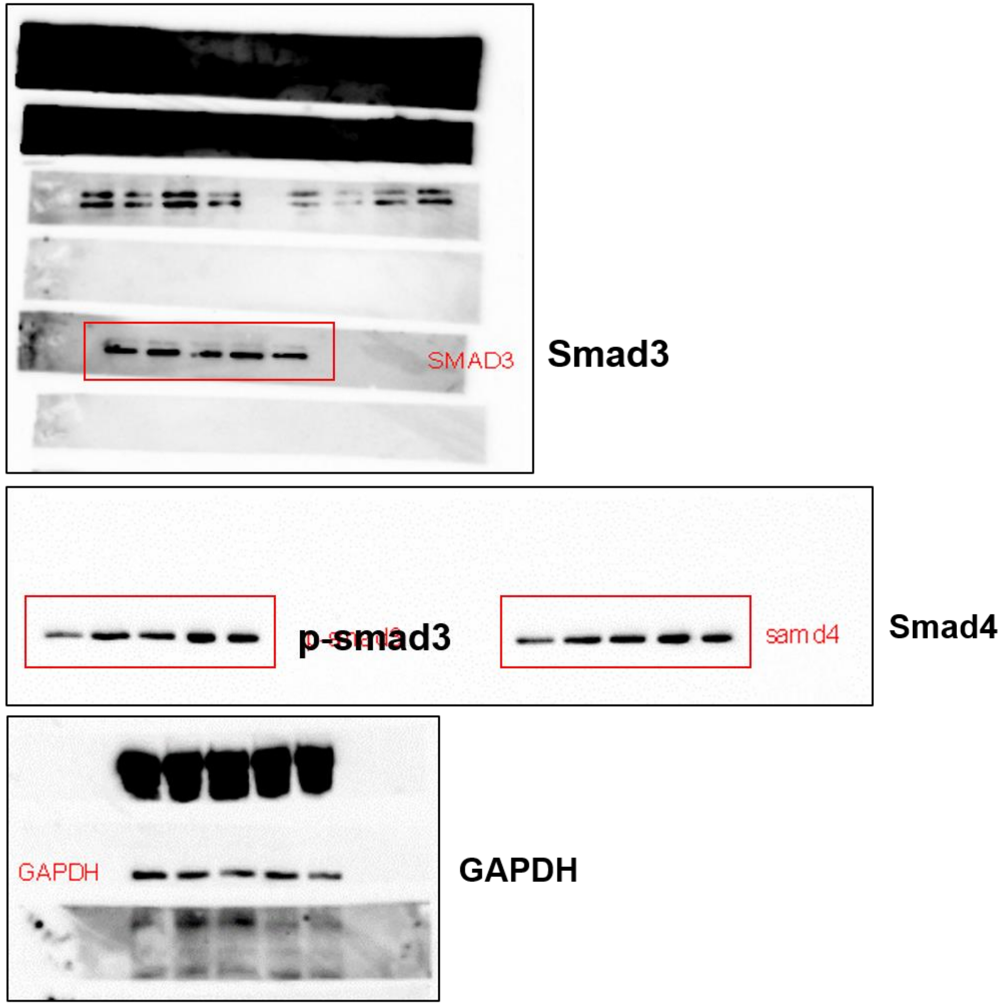
SMAD3
Smad3
p-smad3
samd4
Smad4
GAPDH
GAPDH

Fig.5B

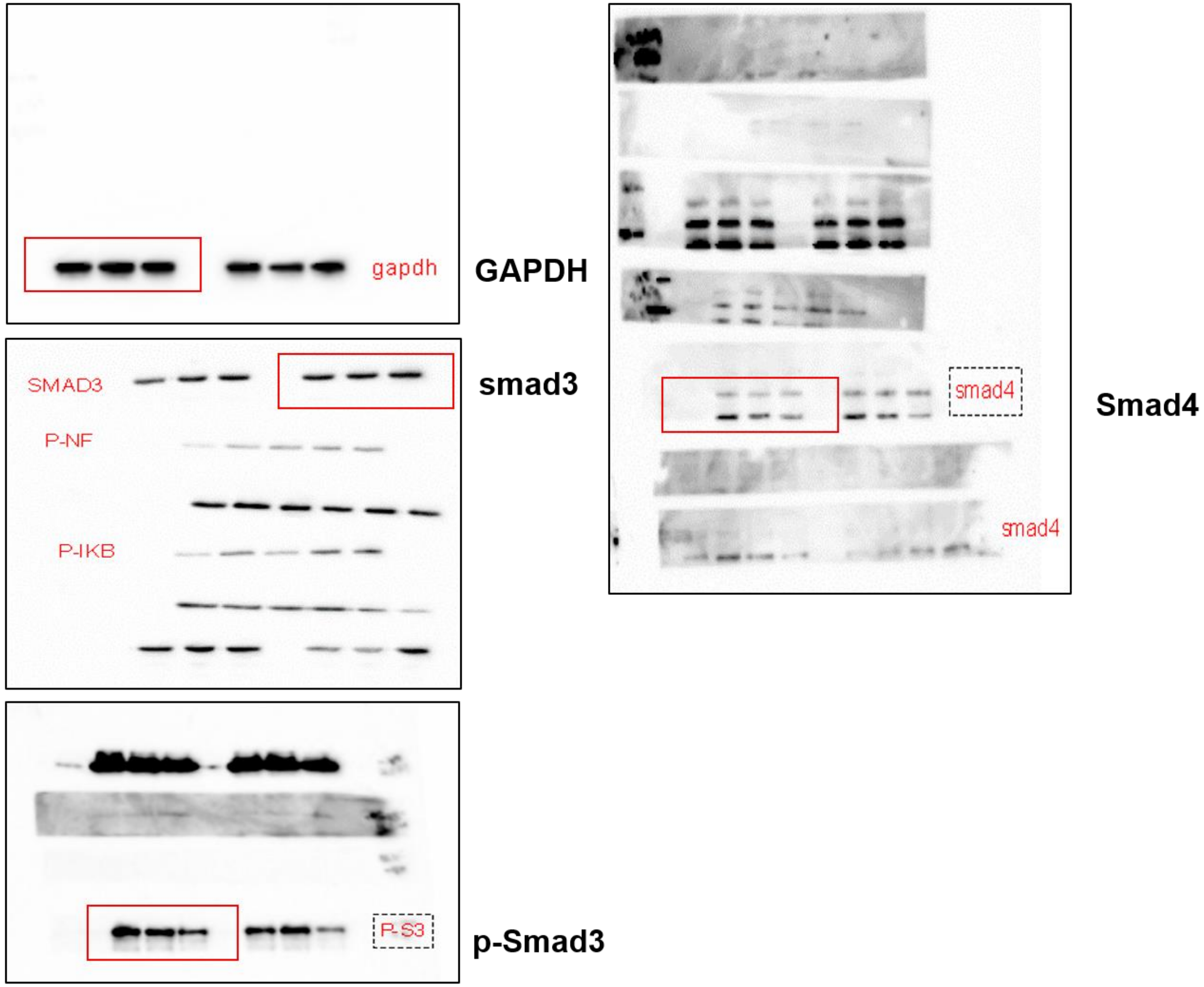
gapdh
GAPDH
SMAD3
smad3
P-NF
P-IKB
P-S3
p-Smad3
smad4
Smad4
smad4

Fig.5C

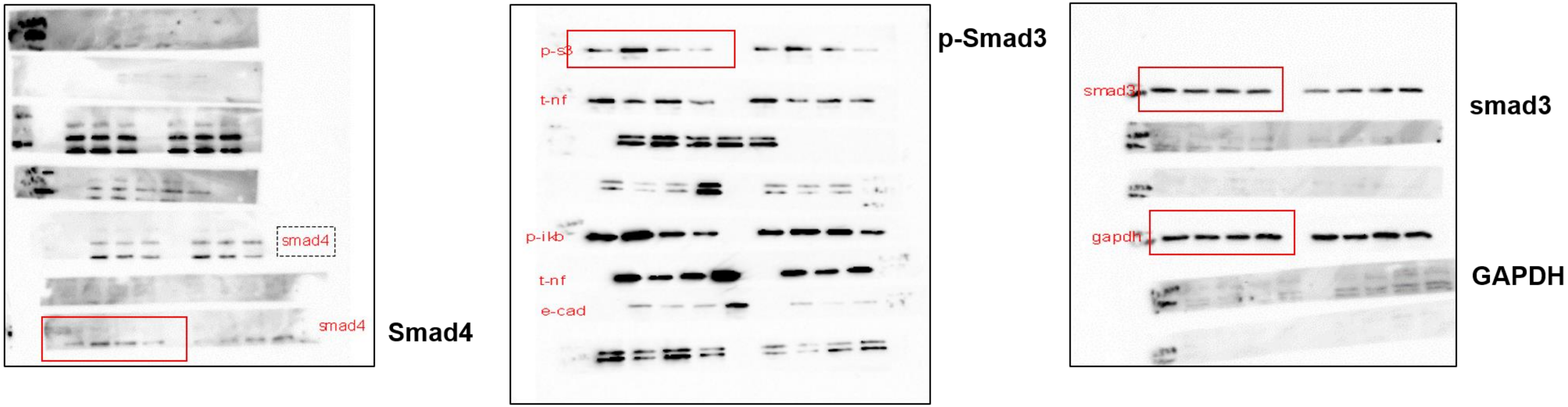

smad4
smad4
Smad4
p-s3
t-nf
p-ikb
t-nf
e-cad
p-Smad3
smad3
gapdh
smad3
GAPDH

Fig.6A

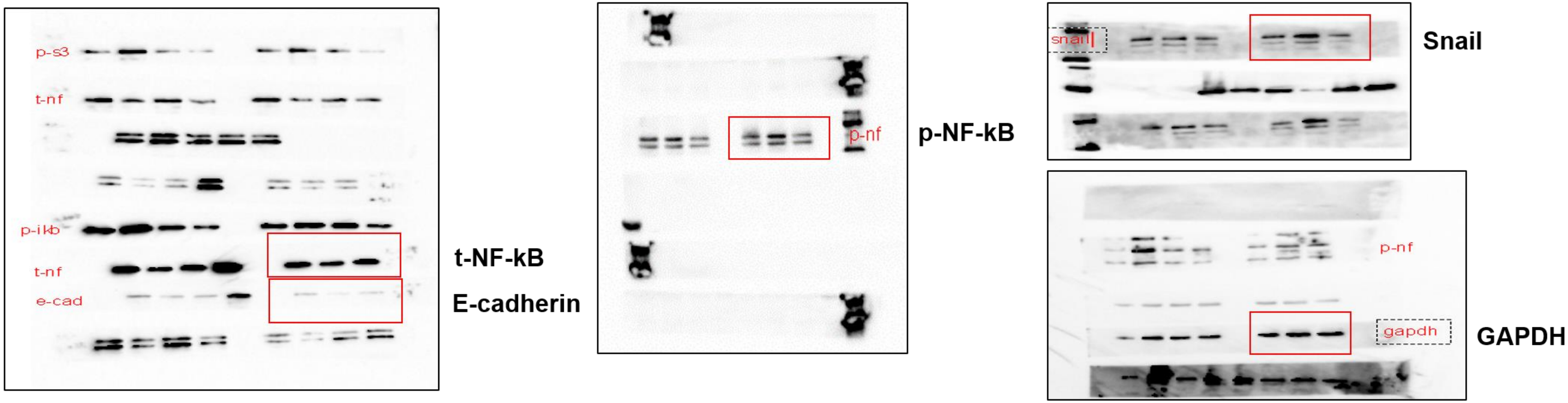
p-s3
t-nf
p-ikb
t-nf
e-cad
t-NF-kB
E-cadherin
p-nf
p-NF-kB
snail
Snail
p-nf
gapdh
GAPDH

Fig.6D

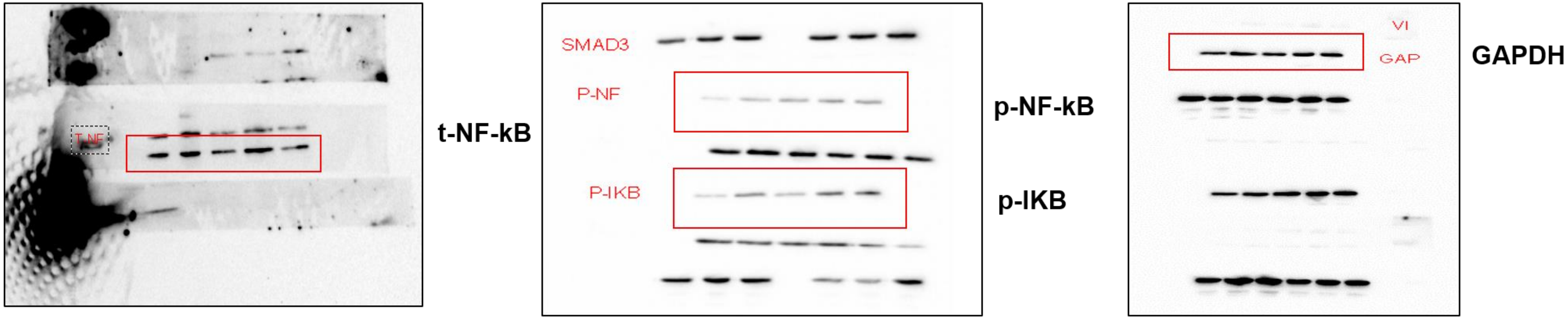
T-NF
t-NF-kB
SMAD3
P-NF
P-IKB
p-NF-kB
p-IKB
VI
GAP
GAPDH

Fig.6E

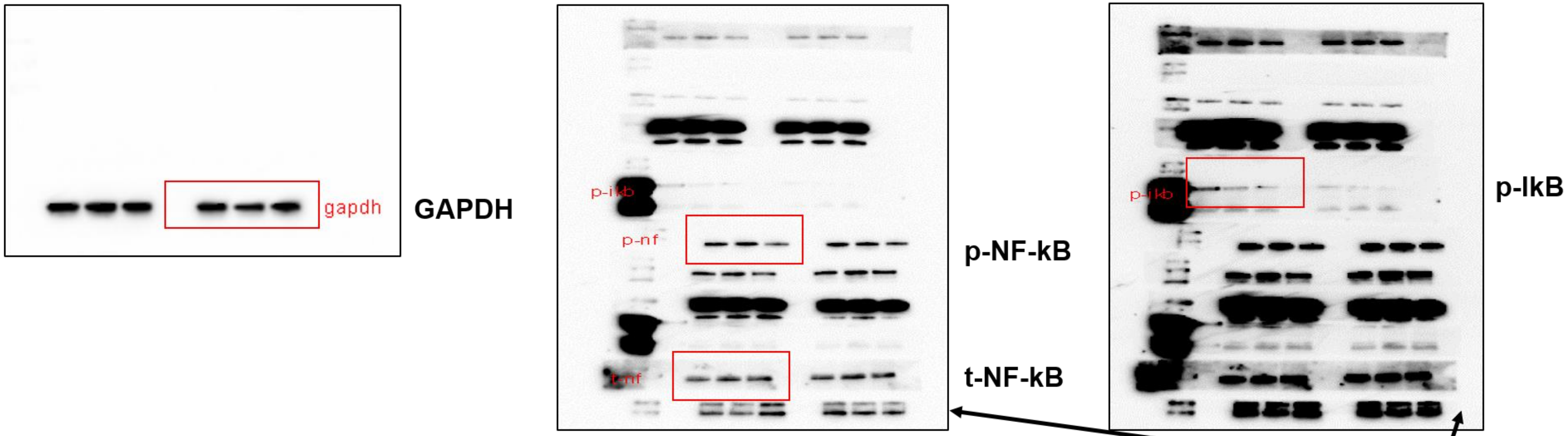
gapdh
GAPDH
p-ikb
p-nf
t-nf
p-NF-kB
t-NF-kB
p-ikb
p-IkB

Fig.6F

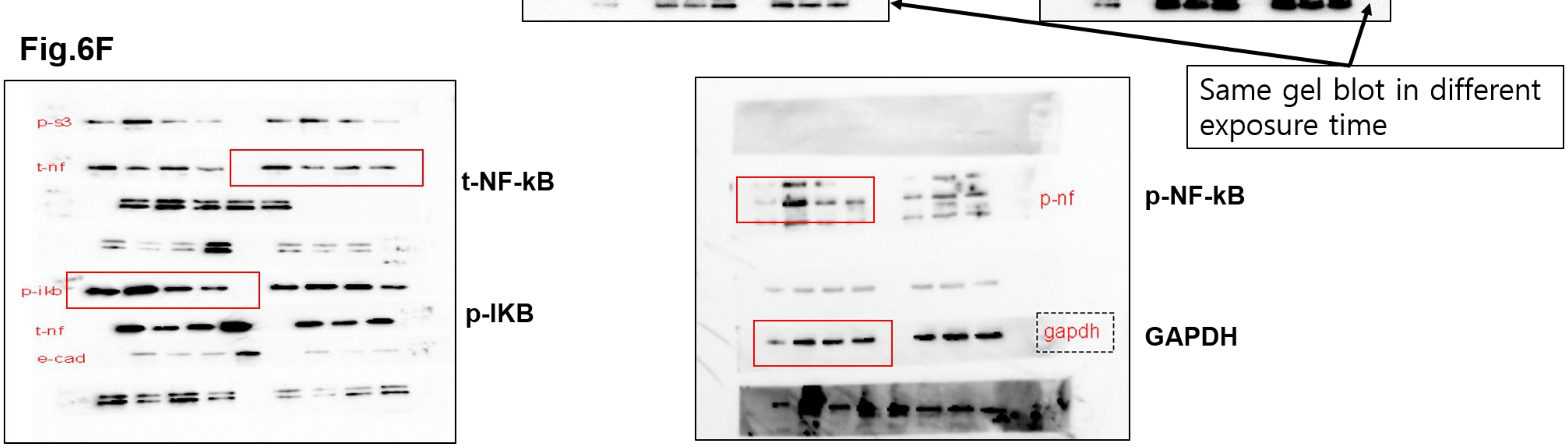
p-s3
t-nf
t-NF-kB
p-ikb
t-nf
e-cad
p-IKB
p-nf
p-NF-kB
gapdh
GAPDH
Same gel blot in different exposure time
